# Pseudogene MSTO2P Interacts with miR-128-3p to Regulate Coptisine Sensitivity of Non-Small-Cell Lung Cancer (NSCLC) through TGF-*β* Signaling and VEGFC

**DOI:** 10.1155/2022/9864411

**Published:** 2022-06-26

**Authors:** Minwei Gu, Xinlian Wang

**Affiliations:** ^1^Department of Thoracic Surgery, Affiliated Hospital of Jiangnan University, Wuxi, Jiangsu 214122, China; ^2^Department of Thoracic Surgery, Xishan People's Hospital of Wuxi, Wuxi, Jiangsu 214105, China

## Abstract

**Background:**

Coptisine has been widely used for treating a variety of cancer types. To date, whether pseudogene is implicated in coptisine resistance of NSCLC remains unknown.

**Methods:**

We performed MTT to assess the cell viability of A549 and Calu-1 cells. The transwell assay was used to examine the invasion of cells. TUNEL was used to determine apoptosis.

**Results:**

Our data showed that coptisine treatment suppressed cell viability and invasion of NSCLC cells while contributing to apoptosis. MiR-128-3p negatively regulated MSTO2P. miR-128-3p reverted MSTO2P knockdown-attenuated cell viability and invasion, as well as promoted cell apoptosis of A549 cells. Moreover, we identified TGF-*β* signaling and VEGFC as key downstream effectors for MSTO2P and miR-128-3p in A549 cells. MiR-128-3p mimic inhibited TGF-*β* pathway-associated genes (TGFBR1, Smad2, Smad5, and Smad9), whereas miR-128-3p inhibitor exerted opposite effect. MSTO2P knockdown led to attenuated expression levels of TGFBR1, Smad2, Smad5 and Smad9. VEGFC overexpression greatly rescued miR-128-3p-modulated cell viability, invasion, and apoptosis of A549 cells.

**Conclusion:**

MSTO2P plays a role in coptisine therapy of NSCLC through miR-128-3p. The findings will advance our understanding of NSCLC treatment.

## 1. Introduction

Lung cancer remains one of the most malignant cancer types. About 85% of lung cancers are non-small-cell lung cancers (NSCLC) [1]. The 5-year overall survival rate is around 16% [2]. Although surgery, radiotherapy, and chemotherapy are widely used approaches for treating cancer patients, drug resistance will limit the use of these therapies [3]. Thus, more effective chemotherapeutic agents for NSCLC need to be developed.

In recent years, researchers have found that natural products such as plant extracts can induce apoptosis in NSCLC and have good potential for anti-NSCLC therapeutic applications. Coptisine is one of the components extracted from the Chinese medication Rhizoma coptidis (RC) [4]. In previous reports, coptisine was involved in different biological processes, including antibacterial, antitumor, and lipid-lowering [5, 6]. In NSCLC, coptisine triggered mitochondrial-dependent cell apoptosis and cell cycle arrest [7]. However, the majority of antitumor drugs are eventually resistant to cancer. The present study studied the molecular mechanism concerning coptisine drug resistance.

Pseudogene derives from protein-coding genes, but it has no ability to translate into proteins [8]. Hence, pseudogenes were initially believed to be nonfunctional RNAs [9]. Recently, increasing evidence has shown that pseudogenes could regulate the progression of various tumors [10–12]. The Misato family member 2 (MSTO2P) pseudogene was found to be implicated in gastric cancer [13], lung cancer [14] and HCC [15]. Moreover, Shi et al. demonstrated that MSTO2P enhanced osteosarcoma progression through PD-L1 [16]. In our study, we attempted to investigate the detailed mechanism of MSTO2P-induced NSCLC.

MiRNAs are short noncoding RNAs which have the ability to play roles in cell proliferation, migration, invasion, and metastasis of tumor cells [17]. Commonly, miRNAs exert their effects via targeting 3′-UTRs of downstream genes [18]. Han et al. showed miR-128 induced apoptosis of pancreatic cancer cells by targeting MDM4 [19]. Some groups revealed that miR-128-3p suppressed breast or colorectal cancer [20, 21]. Besides, miR-128-3p could also promote lung cancer cell apoptosis. However, whether miR-128-3p could be regulated by MSTO2P participating in NSCLC development was obscure.

Transforming growth factor-*β* (TGF-*β*) comprises three isoforms TGF-*β*1, TGF-*β*2, and TGF-*β*3 [22]. These ligands could activate TGF-*β* receptor (TGFBR) to induce SMAD and target genes transcription, thereby regulating tumorigenesis and metastasis [23]. TGFBR1 inhibitor greatly suppressed TGF-*β* signaling [24]. Notably, TGF-*β* pathway was shown to contribute to lung cancer development [25].

The aim of our research is to figure out how pseudogene MSTO2P modulates NSCLC treatment by coptisine. Our results identify miR-128-3p/TGF-*β*/VEGFC as a key regulatory axis for MSTO2P in NSCLC cells.

## 2. Materials and Methods

### 2.1. Cell Culture

The human embryonic kidney cell line 293T and human NSCLC cell lines (A549 and Calu-1) were from the Cell Bank of the Chinese Academy of Sciences. All cells were cultured in complete medium. The complete medium was composed of Dulbecco's Modified Eagle Medium (DMEM) supplemented with 10% FBS and 1% penicillin/streptomycin at 37°C, 5% CO_2_. 25 *μ*g/mL of coptisine (MedChemExpress, HY-N0430) was used to treat the NSCLC cells [26].

### 2.2. Transfection

We used lipo3000 reagent (Invitrogen) to transfect si-MSTO2P, miR-128-3p mimic (30 *μ*M) or inhibitor (30 *μ*M) into A549 and Calu-1 cells. Sequences of these oligonucleotides were listed as follows (5′⟶3′): si-NC: AACGGUUAUCCAGGUUCGG; si-MSTO2P: GCUGCUACAAGAUGAAUAU; NC mimic: AUUGGACCGCAUCGAUCG; miR-128-3p mimic: UCACAGUGAACCGGUCUCUUU; NC inhibitor: CAGCGUUUACCCGGUACGG; miR-128-3p inhibitor: GCCGAUUCAUCCAAGUCAAUCCG.

### 2.3. MTT Assay

We seeded A549 and Calu-1 (∼8000 cells) cells into 96-well plates. Then, the seeded plates were incubated for indicated time. MTT was added and subsequently incubated for 4 h at 37°C. 150 *µ*L DMSO was added into each well and incubated for 10 min. OD570 nm was then measured using a microplate reader.

### 2.4. Invasion Assay

We suspended A549 and Calu-1 cells (2 × 10^5^ cells) in 200 *µ*L of medium. Then, the cell suspensions were placed into the top chamber with an 8 *μ*m pore membrane precoated with Matrigel. Media plus 20% FBS was added to the bottom chamber. About 24 h postinvasion, invaded cells were stained with 0.01% crystal violet. All pictures were captured under a microscope (400x).

### 2.5. TUNEL

We seeded appropriate A549 and Calu-1 cells on the coverslips. The TUNEL reagent was used to stain the cells and the apoptosis rate was determined. DAPI dye was used to stain cell nuclei. All pictures were captured under a microscope (200x).

### 2.6. Reverse Transcription-Quantitative PCR (RT-qPCR)

RNAs of NSCLC cells were extracted with TRIzol reagent (Invitrogen). The total RNAs were dissolved in DEPC ddH_2_O. After that, about 1 *µ*g of total RNA was employed to generate cDNAs using the PrimeScript RT reagent kit (Takara). Real-time PCR was performed using SYBR Green. Relative gene expressions were calculated by 2^−ΔΔCt^ method. Primers used in this study were listed as follows (5′⟶3′): MSTO2P-Forward: GATGGTGTCTGGAGGGTCAA, MSTO2P-Reverse: GCTCTTCCTGGTACTTGGGT. miR-128-3p-Forward: GACTGCCGAGCGAGCG, miR-128-3p-Reverse: GACGCCGAGGCACTCTCTCCT. TGFBR1-Forward: CACAGAGTGGGAACAAAAAGGT, TGFBR1-Reverse: CCAATGGAACATCGTCGAGCA. SMAD2-Forward: ATGTCGTCCATCTTGCCATTC, SMAD2-Reverse: AACCGTCCTGTTTTCTTTAGCTT. SMAD5-Forward: GTGAAGCGATTGTTGGGCTG, SMAD5-Reverse: CAGGTGGCATATAGGCAGGG. SMAD9-Forward: GAAGGCTCCAGGTGTCTCAT, SMAD9-Reverse: GAAGCGGTTCTTGTTGTTTG. VEGFC-Forward: CTCTCTCTCAAGGCCCCAAA, VEGFC-Reverse: AGTCATCTCCAGCATCCGAG. U6-Forward: CCATCGGAAGCTCGTATACGAAATT, U6-Reverse: GGCCTCTCGAACTTGCGTGTCAG. *β*-actin-Forward: TGGCACCACACCTTCTACAA, *β*-actin-Reverse: CCAGAGGCGTACAGGGATAG.

### 2.7. Statistical Analysis

We performed all experiments in triplicate to show the mean ± SD and statistical analysis using Graphpad 6.0. Two or multiple group comparisons were carried out by unpaired Student's *t*-test and ANOVA (Tukey's post hoc test), respectively. *P* < 0.05 was considered as significant.

## 3. Results

### 3.1. Coptisine Inhibited Tumorigenesis of NSCLC Cells

**Figure 1:**
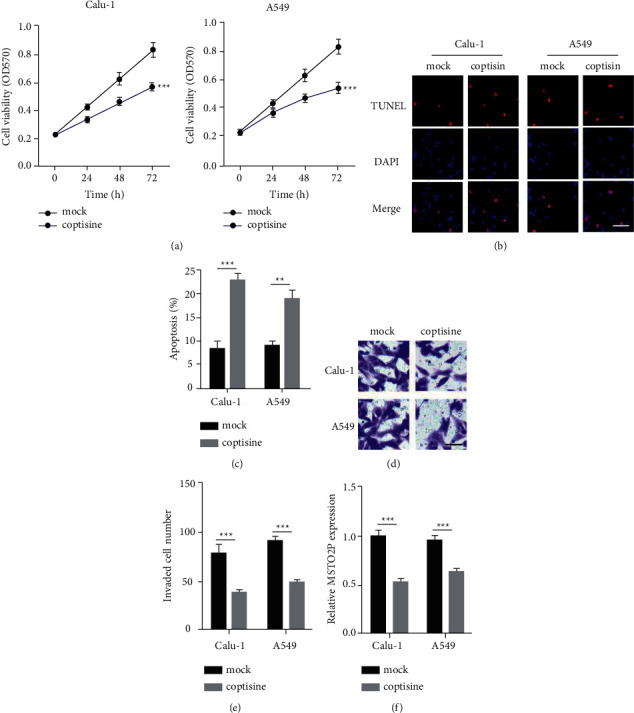
Coptisine inhibited tumorigenesis of NSCLC cells. (a) Cell viabilities were examined by MTT in A549 and Calu-1 cells treated with or without coptisine. ^*∗∗∗*^*P* < 0.001, coptisine group vs. mock group. (b and c) Cell apoptosis of A549 and Calu-1 cells were examined by TUNEL treated with or without coptisine. ^*∗∗*^*P* < 0.01, ^*∗∗∗*^*P* < 0.001, coptisine group vs. mock group. Scale bar = 5 *μ*m. (d and e) Transwell assay showed invasion of A549 and Calu-1 cells treated with or without coptisine. ^*∗∗∗*^*P* < 0.001, coptisine group vs. mock group. Scale bar = 5 *μ*m. (f) MSTO2P levels were measured by RT-qPCR in A549 and Calu-1 cells treated with or without coptisine. ^*∗∗∗*^*P* < 0.001, coptisine group vs. mock group.

To determine the effect of coptisine on NSCLC tumorigenesis, A549 and Calu-1 cells were treated with or without coptisine. First, we examined the cell viability of these two cell lines. The results showed that coptisine significantly inhibited the cell viability of A549 and Calu-1 cells (Figure 1(a)). The TUNEL assay demonstrated that coptisine could increase apoptosis of both A549 and Calu-1 cells (Figures 1(b) and 1(c)). Besides, we performed a transwell assay to determine the invasion ability of NSCLC cells. Coptisine reduced the invaded number of A549 and Calu-1 cells compared to control (Figures 1(d) and 1(e)). Interestingly, we found that coptisine remarkably suppressed pseudogene MSTO2P expression in A549 and Calu-1 cells (Figure 1(f)). Taken together, coptisine markedly inhibited NSCLC tumorigenesis.

### 3.2. MiR-128-3p Regulated MSTO2P Knockdown-Promoted Coptisine Sensitivity of NSCLC

**Figure 2:**
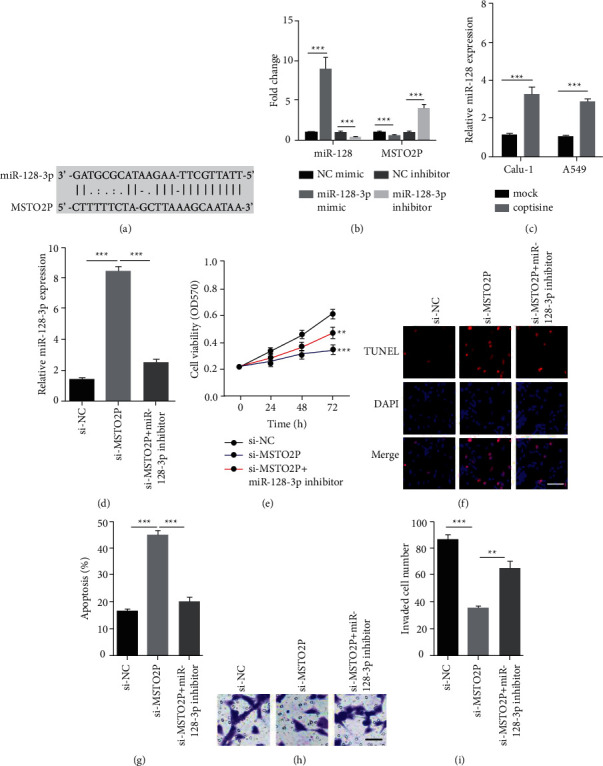
MiR-128-3p regulated MSTO2P knockdown-promoted coptisine sensitivity of NSCLC (a) StarBase 2.0 was used to identify miR-128 as target for MSTO2P. (b) RT-qPCR showed miR-128-3p and MSTO2P levels in A549 cells transfected with NC mimic, miR-128-3p mimic, NC inhibitor, miR-128-3p inhibitor. ^*∗∗∗*^*P* < 0.001, miR-128-3p mimic vs. NC mimic group, miR-128-3p inhibitor vs. NC inhibitor group. (c) RT-qPCR showed miR-128-3p levels in A549 and Calu-1 cells treated with or without coptisine. ^*∗∗∗*^*P* < 0.001, coptisine group vs. mock group. (d) RT-qPCR showed miR-128-3p levels in A549 cells transfected with si-NC, si-MSTO2P, si-MSTO2P plus miR-128-3p inhibitor. ^*∗∗∗*^*P* < 0.001, si-MSTO2P group vs. si-NC group, si-MSTO2P + miR-128-3p inhibitor group vs. si-MSTO2P group. (e) Cell viability was determined by MTT in A549 cells transfected with si-NC, si-MSTO2P, si-MSTO2P plus miR-128-3p inhibitor under coptisine treatment. ^*∗∗*^*P* < 0.01, ^*∗∗∗*^*P* < 0.001, si-MSTO2P group vs. si-NC group, si-MSTO2P + miR-128-3p inhibitor group vs. si-MSTO2P group. (f and g) Cell apoptosis was determined by TUNEL in A549 cells transfected with si-NC, si-MSTO2P, si-MSTO2P plus miR-128-3p inhibitor under coptisine treatment. ^*∗∗∗*^*P* < 0.001, si-MSTO2P group vs. si-NC group, si-MSTO2P + miR-128-3p inhibitor group vs. si-MSTO2P group. Scale bar = 5 *μ*m. (h and i) Cell invasion was determined in A549 cells transfected with si-NC, si-MSTO2P, si-MSTO2P plus miR-128-3p inhibitor under coptisine treatment. ^*∗∗*^*P* < 0.01, ^*∗∗∗*^*P* < 0.001, si-MSTO2P group vs. si-NC group, si-MSTO2P + miR-128-3p inhibitor group vs. si-MSTO2P group. Scale bar = 5 *μ*m.

MSTO2P expression levels were substantially suppressed by coptisine in both A549 and Calu-1 cells. Therefore, we attempted to figure out the underlying molecular mechanism for explaining the role of MSTO2P in NSCLC treated with coptisine. Starbase 2.0 was used to identify miR-128-3p as a putative target of MSTO2P (Figure 2(a)). The RT-qPCR results indicated that miR-128-3p mimic greatly downregulated MSTO2P expression and miR-128-3p inhibitor upregulated MSTO2P expression in A549 cells (Figure 2(b)). Notably, coptisine significantly induced miR-128-3p levels in both A549 and Calu-1 cells (Figure 2(c)). MSTO2P knockdown resulted in elevation of miR-128-3p, which was rescued by transfection of miR-128-3p inhibitor in A549 cells (Figure 2(d)).

Cell viabilities were determined by MTT assay to show that MSTO2P depletion attenuated cell viability compared to si-NC cells, which was partially restored by miR-128-3p inhibitor in A549 cells treated with coptisine (Figure 2(e)). In addition, we measured apoptosis of A549 cells in the presence of coptisine. MSTO2P knockdown evidently induced apoptosis of cells compared with control. The miR-128-3p inhibitor caused a reduction in theapoptosis rate of MSTO2P knockdown cells (Figure 2(f) and 2(g)). Finally, we also observed that MSTO2P knockdown led to impaired invasion ability of A549 cells under coptisine treatment, and this phenomenon was reverted by miR-128-3p inhibitor introduction (Figure 2(h) and 2(i)). In conclusion, our data revealed that miR-128-3p might be critical for MSTO2P-mediated NSCLC tumorigenesis.

### 3.3. MSTO2P and miR-128-3p Regulated TGF-*β* Pathway

**Figure 3:**
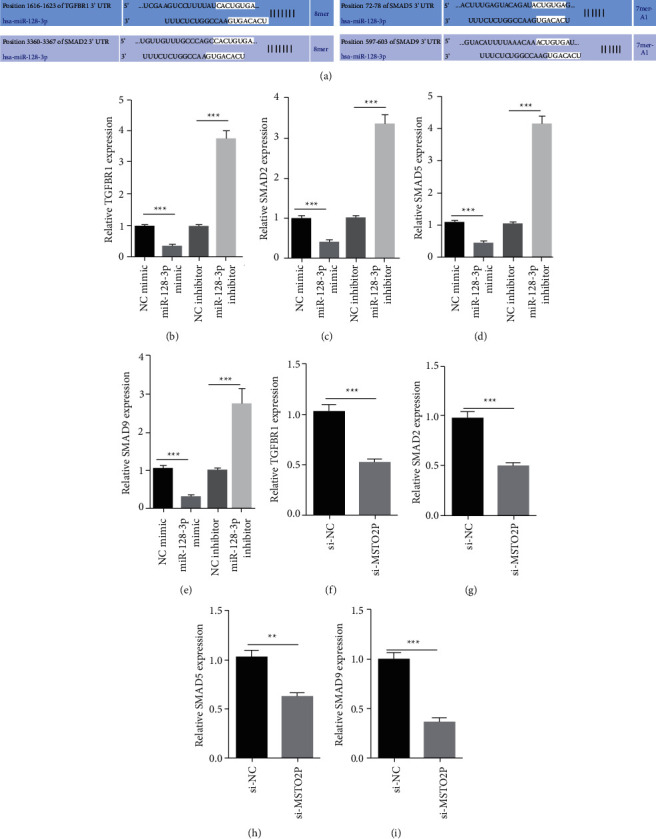
MSTO2P and miR-128-3p regulated TGF-*β* pathway (a) TGFBR1, Smad2, Smad5 and Smad9 were identified as targets of miR-128-3p by TargetScan. (b–e) RT-qPCR showed that TGFBR1, Smad2, Smad5 and Smad9 levels in A549 cells transfected with NC mimic, miR-128-3p mimic, NC inhibitor, miR-128-3p inhibitor. ^*∗∗∗*^*P* < 0.001, miR-128-3p mimic group vs. NC mimic group, miR-128-3p inhibitor group vs. NC inhibitor group. (f–i) RT-qPCR showed that TGFBR1, Smad2, Smad5 and Smad9 levels in A549 cells transfected with si-NC or si-MSTO2P. ^*∗∗∗*^*P* < 0.001, si-MSTO2P group vs. Si-NC group.

To investigate the downstream signaling pathway responsible for MSTO2P/miR-128-3p-mediated tumorigenesis of NSCLC cells, we used TargetScan to identify TGF-*β* pathway as the putative targets for miR-128-3p (Figure 3(a)). Among the targets, expression levels of TGFBR1, Smad2, Smad5 and Smad9 were measured by RT-qPCR in miR-128-3p mimic or miR-128 inhibitor A549 cells. We observed that TGFBR1, Smad2, Smad5 and Smad9 were markedly decreased by miR-128-3p mimic, whereas these gene expression levels were upregulated by miR-128-3p inhibitor (Figure 3(b)–3(e)). In addition, we also examined expression levels of TGFBR1, Smad2, Smad5 and Smad9 in A549 cells transfected with si-NC or si-MSTO2P. The results showed that these gene expressions were downregulated by MSTO2P knockdown (Figure 3(f)–3(i)). In sum, MSTO2P and miR-128-3p modulated TGF-*β* pathway in NSCLC cells.

### 3.4. VEGFC Was a Downstream Effector for MSTO2P/miR-128-3p in NSCLC Cells

**Figure 4:**
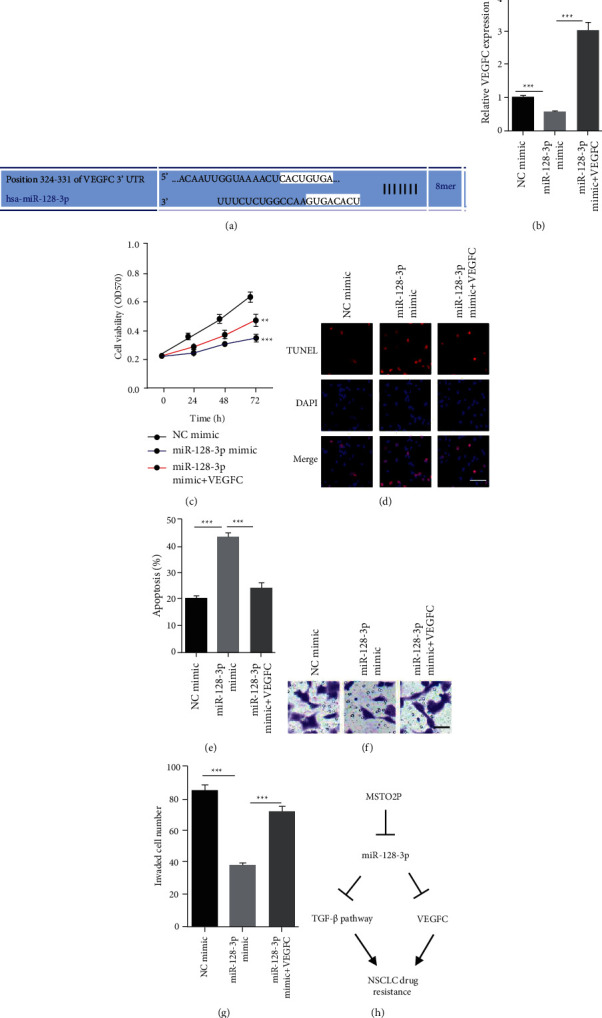
VEGFC was a downstream effector for MSTO2P/miR-128-3p in NSCLC cells (a) VEGFC was identified as target of miR-128-3p by TargetScan. (b) VEGFC levels were determined by RT-qPCR in A549 cells transfected with NC mimic, miR-128-3p mimic, miR-128-3p mimic plus pcDNA3.1-VEGFC. ^*∗∗∗*^*P* < 0.001, miR-128-3p mimic group vs. NC mimic group, miR-128-3p mimic + VEGFC group vs. miR-128-3p mimic group. (c) Cell viability was determined by MTT in A549 cells transfected with NC mimic, miR-128-3p mimic, miR-128-3p mimic plus pcDNA3.1-VEGFC under coptisine treatment. ^*∗∗*^*P* < 0.01, ^*∗∗∗*^*P* < 0.001, miR-128-3p mimic group vs. NC mimic group, miR-128-3p mimic + VEGFC group vs. miR-128-3p mimic group. (d and e) Cell apoptosis was determined by TUNEL in A549 cells transfected with NC mimic, miR-128-3p mimic, miR-128-3p mimic plus pcDNA3.1-VEGFC under coptisine treatment. ^*∗∗∗*^*P* < 0.001, miR-128-3p mimic group vs. NC mimic group, miR-128-3p mimic + VEGFC group vs. miR-128-3p mimic group. Scale bar = 5 *μ*m. (f and g) Cell invasion was determined in A549 cells transfected with NC mimic, miR-128-3p mimic, miR-128-3p mimic plus pcDNA3.1-VEGFC under coptisine treatment. ^*∗∗∗*^*P* < 0.001, miR-128-3p mimic group vs. NC mimic group, miR-128-3p mimic + VEGFC group vs. miR-128-3p mimic group. Scale bar = 5 *μ*m. (h) The working model shows regulation of NSCLC response to coptisine by MSTO2P.

Interestingly, we identify VEGFC as downstream effector for miR-128-3p. VEGFC expression level was determined by RT-qPCR in A549 cells expressing miR-128-3p mimic alone or in combination with VEGFC (Figures 4(a)-4(b)). MTT assay was conducted and showed enhanced cell viability of A549 cells transfected with NC mimic, miR-128-3p mimic, and miR-128-3p mimic plus VEGFC under coptisine treatment. VEGFC could enhance cell viability of miR-128-3p mimic A549 cells (Figure 4(c)). On the contrary, miR-128-3p mimic induced apoptosis of A549 cells under coptisine treatment, which was reverted by VEGFC (Figures 4(d)–4(e)). Besides, we found that VEGFC also rescued the miR-128-3p-attenuated invasion ability of A549 cells under coptisine treatment (Figures 4(f)–4(g)). Taken together, our data revealed that VEGFC acted as a key effector for MSTO2P/miR-128-3p in NSCLC cells (Figure 4(h)).

## 4. Discussion

Advances in chemotherapy of NSCLC have been achieved by using other targeted drugs, such as crizotinib, cetuximab, and bevacizumab [26]. However, few studies reported that natural bioactive components were used to treat NSCLC. In our research, we evaluated the cell viability, invasion, and apoptosis of A549 and Calu-1 cells under coptisine treatment. The results showed coptisine significantly inhibited cell viability and invasion. Based on these, we reckoned that coptisine might be a useful strategy for NSCLC treatment.

Pseudogene has been shown to regulate tumor phenotypes via affecting transcriptional or post-transcriptional processes [27]. Unfortunately, the precise mechanisms of this regulation have not been uncovered. Liao et al. demonstrated that pseudogene LGMN sponged miR-495-3p to promote glioblastoma progression [28]. DUXAP8 promoted pancreatic cancer cell growth by epigenetically regulating CDKN1A and KLF2 [29]. In our paper, we believed that MSTO2P acted as a sponge for miR-128-3p in NSCLC cells.

MiR-128-3p was regarded as a potential target for cancer, because it has been investigated in many cancers [20, 21, 30]. MiR-128-3p played an inhibitory role in EMT of osteosarcoma and induced glioma cell apoptosis [31, 32]. In agreement with these data, we also confirmed miR-128-3p reverted NSCLC cell tumorigenesis-affected by MSTO2P. miR-128-3p inhibited cell viability and invasion of NSCLC cells. Hence, it is possible to use miR-128-3p as a diagnostic and therapeutic marker for NSCLC.

TGF-*β* pathway was involved in NSCLC initiation and metastasis by activating SMAD cascade [33]. Primarily, TGF-*β* binds to TGFBR to phosphorylate SMAD2/3, which then forms complex with SMAD4. The complex will translocate into the nucleus to regulate target gene transcription [34]. In previous papers, some tumor-promoting genes (like Snail1, Slug, ZEB1/2, MMP2, MMP9, and ADAM12) were found to be modulated by TGF-*β* [35]. Consistent with previous studies, our results elucidated that MSTO2P/miR-128-3p regulated coptisine sensitivity of NSCLC cells via TGF-*β* pathway.

The vascular endothelial growth factor (VEGF) belongs to the growth factor family. They are associated with angiogenesis and other cellular events [36, 37]. VEGF is composed of five members: VEGFA, VEGFB, VEGFC, VEGFD, and placental growth factor [38]. They exert their effects via binding to VEGFR1-3. VEGFC binds to VEGFR2 and plays a role in lung, colorectal, and breast cancer cells [39, 40]. Our data revealed that VEGFC remarkably rescued miR-128-3p-attenuated NSCLC phenotypes under coptisine treatment.

In summary, we propose a novel molecular regulatory axis of MSTO2P/miR-128-3p/TGF-*β*/VEGFC in NSCLC. This axis regulates NSCLC response to coptisine. Our conclusions will facilitate the development of combination treatments of coptisine and other targeted therapies.

## Data Availability

Data included in this study are available from the corresponding author upon reasonable request.
